# Practical Observations on Some of the Diseases of the Stomach and Alimentary Canal

**Published:** 1847-07

**Authors:** 


### ART. XIX.

*Practical Observations on some of the Diseases of the Stomach and Alimentary Canal.* By JAMES ALDERSON, M.D., F.R.S., late Senior Physician to the Hull Infirmary.—*London*, 1847. 8vo, pp. 208. With Ten coloured Plates.

THE author of this volume, after having long enjoyed an extensive practice in the country, has recently withdrawn from its harassing duties and taken up his abode in the metropolis. In the comparative leisure thus temporarily created he has set himself to review his past experience, and the present volume is the first-fruits of the scrutiny. In a modest preface he tells us that he claims "neither the character of a discoverer nor of a theorist, nor of a proposer of any particularly novel or scientific plan of treatment." He has written, he says, "in the belief, or at least the hope, that the result of extensive observation, classified and reasoned upon, would be found useful." He may rest assured that his professional brethren will give him all credit both for his motives and his exertions, and will receive his book in the honorable spirit in which it is presented to them.

The work is divided into two Parts—the first, containing observations on structural, the second on functional diseases of the alimentary canal. The structural diseases noticed are—carcinoma of the pharynx, œsophagus, stomach, pylorus ; malignant disease of the colon ; encephaloid disease of the liver ; hypertrophy of the stomach ; perforation of the stomach and ileum. The functional diseases are, different forms of dyspepsia, and disorder of the colon.

The work consists mainly of cases with remarks, many of which are well illustrated by engravings. To such of our readers as are familiar with the special works on cancer in general, and with the various illustrated treatises on morbid anatomy, the volume of Dr. Alderson will convey little or no new information : as an authentic record, however, of cases of severe diseases, traced to their termination and beyond, and subjected to rational and enlightened treatment, it will deserve the attention of practitioners as well as of systematic writers and pathologists.

In Chapter VII. entitled "Carcinoma of the stomach, external to it, and involving other organs," we have a very good account of a form of malignant disease which is of frequent occurrence and yet often misunderstood. It is sometimes confounded with enlargement of the liver or spleen, often with the "tubercular accretion" of Dr. Baron, and we have known it confounded with aneurism of the aorta. We extract the brief account given of the pathology of this disease, and also a part of the author's observations on the diagnosis, symptoms and treatment ; and with these extracts we shall close this brief notice.

"Deposit of carcinomatous matter may take place beneath, or on the free surface, of the serous membrane covering the stomach, and the neighbouring viscera (as the duodenum, the head of the pancreas, the omenta, the mesocolon, &c.) : the deposit varies in consistence from the hardness of scirrhus to the softness of pulp. It is difficult, if not impossible, to assign a position to the earlier deposit : as it increases, however, lymph is effused, and adhesions are formed, by which contiguous parts are glued together ; the viscera become bound to the spine, and to each other, by these adhesions, and, as a consequence, the healthy performance of their various functions is interfered with. The adhesions often present a dark smoky hue, and jelly-like appearance, and are easily torn through ; the proximity of organs, rather than the similarity of function, seems to be the cause of their being involved. The order in which the parts are severally included in the diseased mass may be inferred from the succession of the symptoms which will be hereafter detailed ; this order, as well as the number of organs finally included, varies in different cases. In some cases the disease proceeds until portions of the stomach, the duodenum, the pancreas, the colon, and even the liver, are all involved in one diseased mass, forming an undefined tumour. In the later stages of the disease ulceration finds its way into the duodenum, the stomach, or the colon. The ulceration has a peculiar character, differing from that which results when the primary seat of the deposit is in the submucous cellular tissue ; the disorganising process is, perhaps, more properly expressed by erosion, which takes place through the coats, where either hard or soft material of carcinoma is deposited.

"When the extension of the disease is to the duodenum, and perforation of the coats takes place, the orifice of the gall-ducts becomes involved, and jaundice supervenes; the caliber of the intestine becomes narrowed, and the stomach in consequence obtains a habit of unusual distension.

"The pancreas is ascertained generally by post-mortem examination to be included in the disease; and from its proximity it is to be inferred that it is simultaneously affected with the duodenum: there is no train of symptoms, however, with which I am acquainted to give certain notice of this organ being involved.

"When the extension of the disease is towards the stomach, similar erosion of the coats takes place as in the duodenum, and is followed by the same set of symptoms as have been enumerated as denoting ulceration of the mucous coat.

"When the extension is to the colon, the centre of the arch, from proximity, is the part attacked; the bowel at that point becomes narrowed, and there is a mechanical obstruction to the passage of the antecedent contents; the contractile power of the muscular coat is at the same time interfered with by the adhesions: the capacity of the whole tube to this point enlarges, and the bowel remains habitually distended, just as the stomach does by pyloric or duodenal interference. This state of the colon gives rise to the accumulation which will be noticed as a prominent symptom; diarrhœa or dysentery follow from the effects of ulceration through the mucous lining of the colon.

"The liver is secondarily affected by the deposit of encephaloid tubera, and in some cases subsequently becomes adherent to the diseased mass; the gall-bladder is usually distended with bile, and contains gall-stones. Secondary deposit also sometimes takes place in the lungs, though very rarely, but it does not proceed to a state to cause any very prominent pulmonic symptoms." (pp. 96-9.)

"Amongst the earlier symptoms of this disease, unsatisfactory relief from the bowels is the most prominent. It is experienced for a year or more before the more urgent symptoms set in, and is accompanied at intervals by nausea, retchings, and headache. Pain is also suffered, but not to any great extent, and it is referred by the patient to an unremoved accumulation in the bowels; it is described as situated at the pit of the stomach, or rather lower. The patient subsequently begins to lose flesh, the features shrink, the complexion becomes opaque, sallow, and exsanguineous; the eye looks sunken, and the strength begins to fail: great anxiety about his health now possesses the patient, who is often prone to seek various opinions, until he finds a voice ready to flatter him with the delusive promise of a cure." (p. 100.)

"Within three or four months of a fatal termination a careful examination of the abdomen usually reveals an unusual fulness a little to the right of the pit of the stomach; it is hardly felt as a tumour, but as an undefined resisting mass, and it appears to rest upon the spine: pressure by the hand causes pain; and, the same as in cases of ulcerated carcinoma of the stomach, I have several times noticed that the patient exclaims with satisfaction that the precise seat of the pain has been touched, and he generally adds his conviction that the cause will be removed. At this time loss of flesh and strength increases, the appetite fails entirely, there are vomitings and retchings: the nights are restless, attended with pain. Hiccup is a very distressing symptom, and increases both in violence and in duration of the fit as the disease advances." (p. 101.)

"It is obvious that, according to the organs included in the disease, their different functions will be interfered with. Amongst the most constant symptoms of the first stage of the complaint is the sensation of load in the bowels. Relief is only obtained by several daily evacuations, and the uneasy sensations again daily return. This partial relief may arise from the actual removal of a lodgment of feculent matter in the arch of the colon, or by the aperient acting as an antiphlogistic measure, by causing increased secretion from the mucous coat: but I am inclined to think, from the fact that the uneasy sensations recur again so soon after copious action of the bowels, that the uneasiness is rather to be attributed to the presence of the carcinomatous deposit, and its progressing adhesions, than to accumulation within the bowel, to which the patient always attributes it.

"In every stage of the complaint the evacuations will give evidence of there having been accumulation in the colon, which will seem to call for the repetition of purgatives; but as the difficulty to be overcome is, after all, merely a symptom, it is necessary to be cautious in subjecting the patient to a severe discipline which could only be warranted on the supposition that the endurance would be rewarded by permanent benefit." (pp. 111-3.)

"At an early period of the disease the deposit is evidently confined to a small space; the future ramifications have yet to be determined; and the symptoms obviously vary according to the progress of the encroachment: thus hepatic symptoms become more prominent as the disease proceeds towards the duodenum and pylorus. Mechanical opposition to the food from the stomach begins, and vomiting comes on. The vomiting is not of the same kind as that in cases of ulceration of the mucous lining; it is full and free, as in scirrhous pylorus: the stomach becomes habitually distended, and admits of a large accumulation of contents, which are sometimes retained for a period of twenty-four hours or more, and then rejected all at once, without any accompanying nausea. The stomach, being comparatively in a sound state, craves for food immediately after it has got rid of the contents, and hence the voracious appetite which is sometimes experienced. The eagerness for food is also to be attributed to a sense of relief obtained by a full state of the organ which replaces the neighbouring viscera in their easiest relative position: in such a case, it is impossible to deny the patient the solid food which he desires." (pp. 115-6.)

---

